# *TBC1D32* variants disrupt retinal ciliogenesis and cause retinitis pigmentosa

**DOI:** 10.1172/jci.insight.169426

**Published:** 2023-11-08

**Authors:** Béatrice Bocquet, Caroline Borday, Nejla Erkilic, Daria Mamaeva, Alicia Donval, Christel Masson, Karine Parain, Karolina Kaminska, Mathieu Quinodoz, Irene Perea-Romero, Gema Garcia-Garcia, Carla Jimenez-Medina, Hassan Boukhaddaoui, Arthur Coget, Nicolas Leboucq, Giacomo Calzetti, Stefano Gandolfi, Antonio Percesepe, Valeria Barili, Vera Uliana, Marco Delsante, Francesca Bozzetti, Hendrik P.N. Scholl, Marta Corton, Carmen Ayuso, Jose M. Millan, Carlo Rivolta, Isabelle Meunier, Muriel Perron, Vasiliki Kalatzis

**Affiliations:** 1 Institute for Neurosciences of Montpellier (INM), University of Montpellier, Inserm, Montpellier, France.; 2 National Reference Centre for Inherited Sensory Diseases, University of Montpellier, CHU, Montpellier, France.; 3 Université Paris-Saclay, CNRS, Institut des Neurosciences Paris-Saclay, Saclay, France.; 4 Institute of Molecular and Clinical Ophthalmology Basel (IOB), Basel, Switzerland.; 5 Department of Ophthalmology, University of Basel, Basel, Switzerland.; 6 Department of Genetics and Genome Biology, University of Leicester, Leicester, United Kingdom.; 7 Department of Genetics, Health Research Institute-Fundación Jiménez Díaz University Hospital, Universidad Autónoma de Madrid (IIS-FJD, UAM), Madrid, Spain.; 8 Center for Biomedical Network Research on Rare Diseases (CIBERER), Instituto de Salud Carlos III, Madrid, Spain.; 9 Molecular, Cellular and Genomics Biomedicine Research Group, Instituto de Investigación Sanitaria La Fe (IIS La Fe), Valencia, Spain.; 10 Joint Unit of Rare Diseases, IIS La Fe-Centro de Investigación Príncipe Felipe, Valencia, Spain.; 11 Department of Neuroradiology and; 12 Institute for Human Functional Imaging (I2FH), University of Montpellier, CHU, Montpellier, France.; 13 Department of Medicine and Surgery,; 14 Department of Medical Genetics,; 15 Department of Nephrology, and; 16 Neuroradiology Unit, Diagnostic Department, University Hospital of Parma, Parma, Italy.

**Keywords:** Genetics, Ophthalmology, Genetic diseases, Retinopathy, iPS cells

## Abstract

Retinitis pigmentosa (RP) is the most common inherited retinal disease (IRD) and is characterized by photoreceptor degeneration and progressive vision loss. We report 4 patients presenting with RP from 3 unrelated families with variants in *TBC1D32*, which to date has never been associated with an IRD. To validate *TBC1D32* as a putative RP causative gene, we combined *Xenopus* in vivo approaches and human induced pluripotent stem cell–derived (iPSC-derived) retinal models. Our data showed that *TBC1D32* was expressed during retinal development and that it played an important role in retinal pigment epithelium (RPE) differentiation. Furthermore, we identified a role for TBC1D32 in ciliogenesis of the RPE. We demonstrated elongated ciliary defects that resulted in disrupted apical tight junctions, loss of functionality (delayed retinoid cycling and altered secretion balance), and the onset of an epithelial-mesenchymal transition–like phenotype. Last, our results suggested photoreceptor differentiation defects, including connecting cilium anomalies, that resulted in impaired trafficking to the outer segment in cones and rods in *TBC1D32* iPSC-derived retinal organoids. Overall, our data highlight a critical role for TBC1D32 in the retina and demonstrate that *TBC1D32* mutations lead to RP. We thus identify *TBC1D32* as an IRD-causative gene.

## Graphical abstract

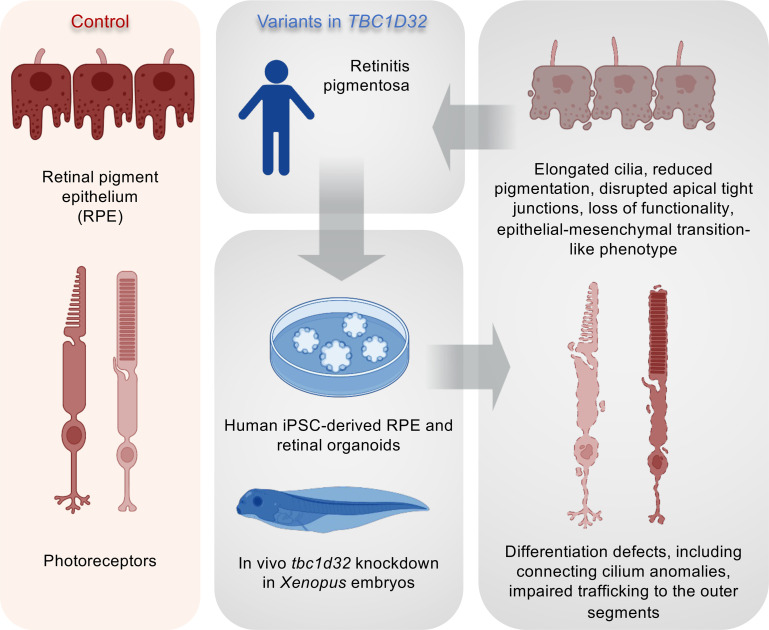

## Introduction

Inherited retinal diseases (IRDs) are a group of clinically and genetically heterogeneous disorders characterized by a dysfunction or degeneration of the light-sensing cells of the retina, the photoreceptors, and/or their underlying support tissue, the retinal pigment epithelium (RPE) (1). In the majority of cases, IRDs are associated with a progressive loss of vision that can have a variable age of onset and severity of symptoms. Although each disease is monogenic, there are more than 270 known causative genes (RetNet, https://sph.uth.edu/retnet). The most common IRD is a rod-cone dystrophy known as retinitis pigmentosa (RP) (2), characterized by night blindness appearing in childhood and followed by a progressive loss of peripheral vision. There are more than 90 causative RP genes with a collective prevalence of 1 in 4,000 (3) and with autosomal-dominant, autosomal-recessive, or X-linked modes of transmission (4). Furthermore, mutations in the same gene can give rise to either isolated RP or RP associated with extra-retinal symptoms.

The clinical heterogeneity associated with RP is partly explained by the diverse localizations and roles of the proteins encoded by the causative genes within the retina (5). The photoreceptors have a unique morphology comprising an axon, nucleus-containing cell body, mitochondria-rich inner segment (IS), and outer segment (OS) filled with lipid disks where the phototransduction process is initiated following light interaction. The IS is connected to the OS by a highly modified primary cilium called the connecting cilium (CC). The genes responsible for isolated RP can be divided into groups that play key roles in the cell body (splicing, transcription, development), IS (transport, signaling) or OS (structure, phototransduction) (3, 5). The genes for syndromic RP are mostly localized at the level of the CC or the periciliary membrane complex (6). These diseases are also known as ciliopathies, and although they can give rise to isolated RP, retinal defects are often linked to defects in other ciliated tissues (7). Interestingly, the RPE contains a primary cilium on its apical side, which is critical for RPE maturation and homeostasis maintenance (8), and RPE ciliary defects were recently associated with retinal ciliopathies (9).

Currently, the genetic causes in approximately 40% of patients with RP remain unknown (4). To resolve such cases, we performed whole-exome sequencing (WES) in the probands from 3 unrelated families presenting clinically with autosomal-recessive RP and identified biallelic variants in *TBC1D32* (OMIM: 615867). TBC1D32 belongs to a family of Rab GTPase-activating proteins (GAPs) containing a Tre-2, Bub-2, and Cdc16 (TBC) domain. TBC1D32 has been shown to have a role in regulating the structure of the primary cilium in the neural tube (10). Zebrafish *tbc1d32* morphants showed a curvature of the body axis and hydrocephalus, and a *TBC1D32*-knockout mouse model exhibited neural patterning defects comprising exencephaly, preaxial polydactyly, and poorly developed eyes. Accordingly, recessive loss-of-function *TBC1D32* variants were reported as responsible for oral-facial-digital (OFD) syndrome type IX (OMIM: 258865), a ciliopathy characterized by defects in development of the oral cavity, face, and digits, associated with microphthalmia/anophthalmia (11). However, OFD syndrome is not classically associated with retinal anomalies, and the *TBC1D32* animal models do not have reported retinal defects.

Therefore, to validate *TBC1D32* as a putative RP causative gene, we combined animal and human disease modeling by (*i*) performing hypomorphic knockdowns in an in vivo *Xenopus* model and (*ii*) generating induced pluripotent stem cell–derived (iPSC-derived) RPE and retinal organoids from a patient with RP carrying *TBC1D32* variants. Together, our data show that *TBC1D32* is expressed during retinal development and plays an important role in ciliogenesis. The disruption of *TBC1D32* results in abnormal RPE maturation and function and defective photoreceptor differentiation and OS trafficking. Overall, we identify *TBC1D32* as an IRD-causative gene, which has implications for alleviating current diagnostic deadlocks.

## Results

### Three unrelated families present with clinical features of RP.

**Figure 1:**
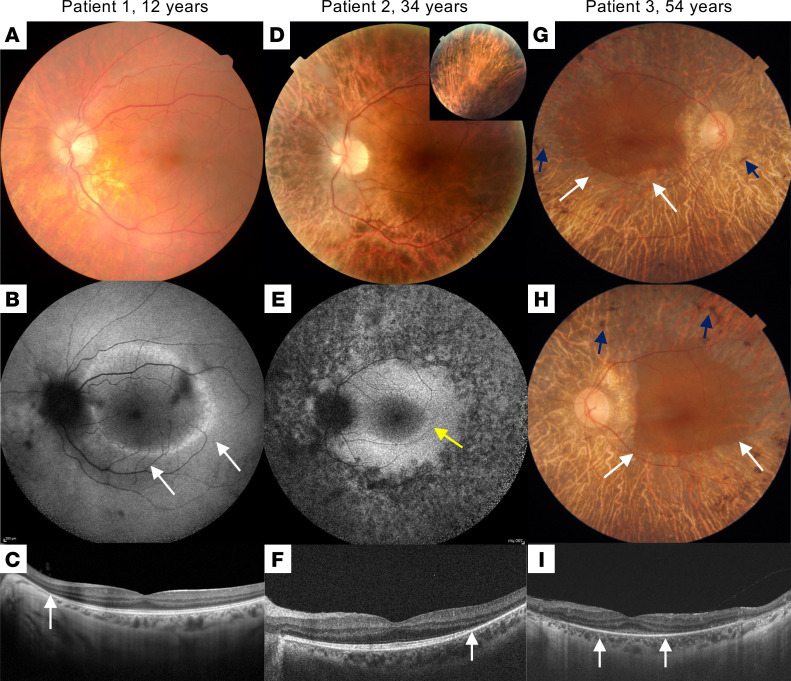
Multimodal imaging of the patients. Patient 1: (**A**) Color fundus photograph showing pallor of the optic nerve head and reduced caliber of the retinal vessels. Note the abnormal visibility of the choroidal vessels due to RPE damage in the midperiphery. (**B**) Short-wavelength fundus autofluorescence (SW-FAF) showing a parafoveal ring characteristic of RP (white arrows). (**C**) SD-OCT macular scan showing preserved segmentation in the foveal area with peripheral loss of the ellipsoidal zone (EZ) line nasal to the fovea (white arrow). Patient 2: (**D**) Color fundus photograph showing increased choroidal visibility beyond the temporal vascular arcades. Inset: pigment accumulation in the superior retinal periphery. (**E**) SW-FAF showing a relatively preserved intensity within the macular region and loss of the signal beyond the temporal vascular arcades. An incomplete hyperautofluorescent ring is visible in the temporal parafoveal region (yellow arrow). (**F**) SD-OCT scan along the horizontal meridian through the fovea showing preservation of retinal layers in the central macula and gradual disappearance of photoreceptor layers with increasing eccentricity in the temporal direction. The white arrow indicates the beginning of the disappearance of the EZ line. Patient 3: (**G** and **H**) Color fundus imaging of both eyes showing peripheral retinal bone spicules (blue arrows), papillary pallor, and evident atrophy of the RPE-choriocapillaris complex except in the macular area (delineated by white arrows). (**I**) SD-OCT macular scan showing an EZ line only visible beneath the fovea (between white arrows).

Four patients from 3 unrelated families (patient 1, family 1; patient 2, family 2; patients 3 and 4, family 3) were studied (Supplemental Figure 1; supplemental material available online with this article; https://doi.org/10.1172/jci.insight.169426DS1), and their ocular data are summarized in Supplemental Table 1. Night blindness occurred in childhood (patients 1 and 2) or in early adulthood (patients 3 and 4). Refraction errors were highly variable; the 2 patients older than 50 years were legally blind. Peripheral visual field impairment occurred early in the second decade, with a severe impairment after the age of 50 years. Beyond the classic signs of RP noted on multimodal imaging and on spectral-domain optical coherence tomography (SD-OCT), all patients shared abnormal visibility of large choroidal vessels in the entire peripheral retina, suggesting RPE loss with photoreceptor degeneration (Figure 1). None of the patients had documented macular edema. On full-field electroretinogram recordings, none of the patients had discernable rod and cone responses (data not shown). The patients did not mention any systemic symptoms, and consistently a systemic checkup only revealed mild MRI anomalies with an atrophic aspect of the superior vermis in patients 1 and 2 (Supplemental Figure 2). In addition, patient 2 was diagnosed at 27 years of age with nonproteinuric chronic kidney disease. Urinary protein-to-creatinine and albumin-to-creatinine ratios were 0.19 and 0.10 mg/mg, respectively, with no notable urinary sediment. Autoimmunity markers were negative, and C3 and C4 complement fractions, and IgG, IgA, and IgM plasmatic levels, were normal. Kidneys were bilaterally reduced (95 mm) and showed mild cortical hyperechogenicity with a few bilateral cortical and parapelvic cysts (maximum 9 mm). Polycystic kidney disease (PKD) was ruled out by negative genetic testing of the causative genes *PKD1* and *PKD2*.

### TBC1D32 pathogenic variants segregate with RP in 3 families.

Trio WES was performed on patient 1 and his healthy parents (Supplemental Figure 1A). No mutation in genes previously associated with syndromic and nonsyndromic RP (https://sph.uth.edu/retnet/) was detected. We identified 2 variants in the *TBC1D32* gene (NM_001367760.1) in *trans* in patient 1, 1 of which was carried by each parent. The maternal intron 2 variant c.317+5G>A was predicted to probably alter splicing according to Human Splicing Finder and described at a frequency of 0.000004092 in the Genome Aggregation Database (gnomAD) (https://gnomad.broadinstitute.org/). To ascertain pathogenicity, we performed reverse transcription PCR (RT-PCR) amplification of exons 1 to 5 on whole blood cells (WBCs) and detected 2 bands in the proband and maternal samples, the smaller of which corresponded to in-frame skipping of exon 2 (Supplemental Figure 1D). The paternal variant, c.846delTCCTA; p.(Asn282Lysfs*7) (exon 7), was absent from the gnomAD and was predicted to induce a frameshift and the introduction of a premature termination codon (PTC).

WES performed for patient 2 (Supplemental Figure 1B) showed that he was compound heterozygous for the variants c.18_27del; p.(Ser6Argfs*8) in exon 1 (maternal allele) and c.1141-1G>A in intron 10 (paternal allele). Both variants were absent from databases. The second variant was predicted to strongly alter splicing according to an in silico prediction software (https://mobidetails.iurc.montp.inserm.fr/MD). To confirm its pathogenicity, we performed RT-PCR amplification of exons 9 to 13 on WBCs of the patient and his father and detected as many as 7 additional splicing events that bore PTCs and were not present in the control (Supplemental Figure 1E).

Trio WES was performed on patients 3 and 4 and a healthy sister (Supplemental Figure 1C). The affected siblings were compound heterozygous for the variants c.1267G>T; p.(Glu423*) in exon 12, and c.3513G>T; p.(Trp1171Cys) in exon 31, whereas the unaffected sister was heterozygous for the c.3513G>T variant. The frequency in gnomAD of the c.1267G>T and c.3513G>T variants were 0.0000124 (described 3 times in the database) and 0.00000402 (described once), respectively. The missense variant is situated in the carboxy-terminal Rab GAP TBC domain, in a highly conserved position relative to the orthologous proteins (Supplemental Figure 1F). The in silico pathogenicity scores were ambiguous (https://varsome.com/). Structural analysis using AlphaFold showed that the tryptophan at position 1171 was located at the beginning of an alpha helix, and its substitution by a cysteine residue was likely to create a steric hindrance with a tyrosine at position 1019. However, with a Combined Annotation Dependent Depletion pathogenicity score of 31 (https://cadd.gs.washington.edu/), this variant was classed as of unknown significance according to the American College of Medical Genetics and Genomics classification. Taken together, the patients of all 3 families carried biallelic *TBC1D32* variants, of which only 1 allele could be predicted as loss of function.

### tbc1d32 is expressed in the neuroretina and RPE during Xenopus development.

**Figure 2:**
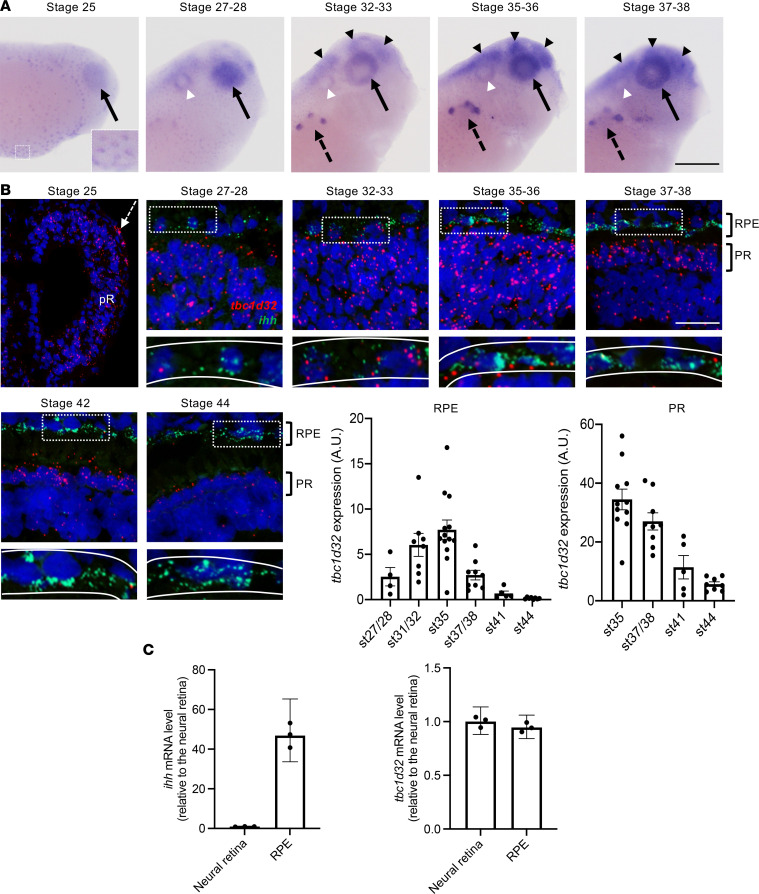
*tbc1d32* expression during *Xenopus* development and retinogenesis. (**A**) Lateral views of *Xenopus* embryo heads (anterior to the right) following *tbc1d32* whole-mount in situ hybridization. Enlargement: at stage 25, epidermal cells are stained. *tbc1d32* expression is also detected in the eye (black arrows) and later in the pronephric nephrostomes (black dotted arrows), brain (black arrowheads), and otic vesicle (white arrowheads). (**B**) RNAscope in situ hybridizations for *tbc1d32* and *ihh* on cryostat sections. A region of RPE cells, delineated by white dotted boxes in the upper panels, is enlarged in the lower panels, where the RPE layer is outlined (white lines). Scatterplots with bars represent the quantification of *tbc1d32* expression in the RPE or photoreceptor layer at each stage (st). Data are represented as mean ± SEM. Each dot represents 1 retina. Scale bars = 400 μm in **A**, 50 μm in **B**. AU, arbitrary units; pR, presumptive retina; RPE, retinal pigment epithelium; PR, photoreceptors. (**C**) qPCR analysis of *ihh* and *tbc1d32* expression in neural retina and RPE tissues, dissected at stage 35–36. *ihh* serves as a specific RPE marker. Data are represented as geometric mean with 95% CI; *n* = 3 technical replicates.

As *TBC1D32* variants were never previously linked to RP, we used a *Xenopus* *laevis* model, which is highly suited to functional development and gene knockdown studies (12), to test the expression and role of *tbc1d32* in the retina. *Xenopus* *tbc1d32* is located on chromosome 5L, displays an exon/intron structure identical to the human homolog (33 exons), and is predicted to encode a 1,297 aa protein with approximately 60% identity to human TBC1D32 (accession NM_001367759.1, NM_001367760.1, or NM_152730.6). We cloned the *Xenopus* *tbc1d32* full-length coding sequence (Supplemental Figure 3) and analyzed its expression during development (Figure 2A). *tbc1d32* transcripts were first detected at stage 25 (13) in multiciliated epithelial cells in the epidermis and in the optic vesicle. From stage 27–28, expression was also detected in the otic vesicle, brain, and pronephric nephrostomes. We further analyzed the expression of *tbc1d32*, and *indian hedgehog* (*ihh*) as an RPE marker (14), on retinal sections using an RNAscope fluorescent multiplex assay (Figure 2B). At stage 25, *tbc1d32* was expressed in all progenitor cells of the optic vesicle. At stage 27–28, transcripts were abundantly detected in the presumptive neural retina, but only a few molecules were detected in the presumptive RPE, labeled with *ihh*. At stages 32–33, *tbc1d32* transcripts were clearly visible in the developing RPE, with a peak at stage 35–36. From stage 37–38 onwards, *tbc1d32* expression decreased in the RPE, inner nuclear, and ganglion cell layers but remained well expressed in the outer nuclear layer (ONL) containing the photoreceptors, where it decreased at later stages. Finally, we verified the presence of *tbc1d32* transcripts in RPE cells by quantitative PCR (qPCR) on mRNAs from dissected RPE at stage 35–36 (Figure 2C). In conclusion, in the retina, *tbc1d32* is expressed in retinal progenitors, developing photoreceptors, and RPE cells, consistent with a putative role in retinogenesis.

### Knockdown of Xenopus tbc1d32 leads to developmental RPE and photoreceptor defects.

**Figure 3:**
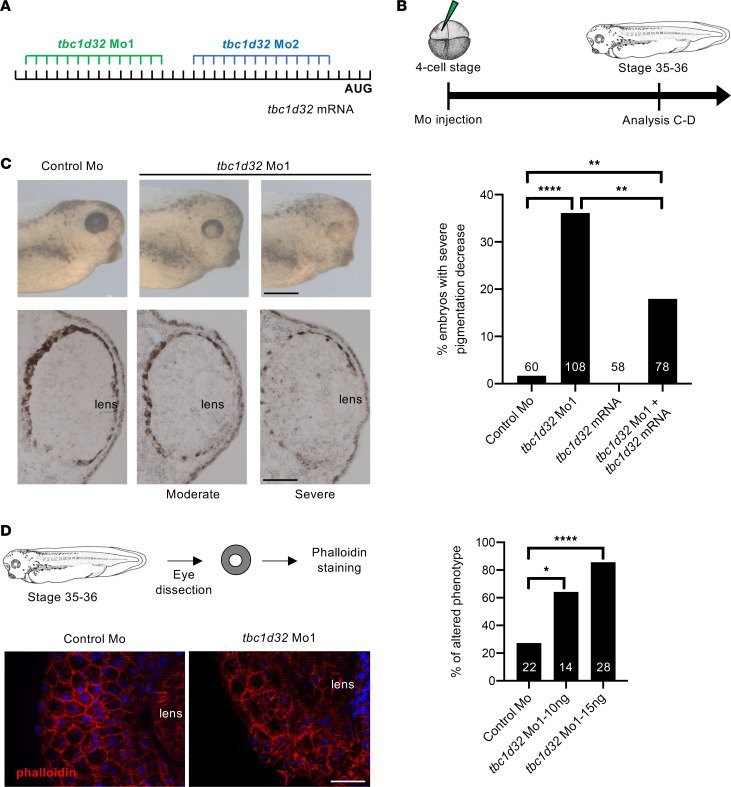
*Xenopus* RPE phenotype following *tbc1d32* knockdown. (**A**) Mo1 and Mo2 target sequences located in the 5′ untranslated region of *tbc1d32* mRNA. (**B**) Diagram of the experimental design. (**C**) Upper panels, lateral views of 1 control and 2 morphant embryo heads with moderate and severe phenotypes (anterior to the right). Lower panels, transverse retinal sections of control and morphant embryos (dorsal side up). The bar plot represents the percentage of embryos with a severe decrease in pigmentation among control (control Mo), morphant (*tbc1d32* Mo1), *tbc1d32* mRNA-injected, and *tbc1d32* Mo1/*tbc1d32* mRNA coinjected groups. The total number of embryos analyzed per condition is indicated in each bar. **P < 0.01; ****P < 0.0001; Fisher’s exact test. (**D**) Phalloidin staining of filamentous actin on dissected eyes of control or morphant *Xenopus* embryos. The bar plot represents the proportion of eyes with an altered distribution of F-actin for each condition. The total number of eyes analyzed per condition is indicated in each bar. *P < 0.05; ****P < 0.0001; Fisher’s exact test. Scale bars = 400 μm for whole mounts and 60 μm for sections in **C**, 20 μm in **D**.

To determine the impact of *tbc1d32* knockdown in retinal development, we designed 2 morpholinos (Mo1 and Mo2) that we injected at the 4-cell stage in 1 blastomere giving rise to neural ectoderm (Figure 3, A and B, and Supplemental Figures 3 and 4). This led to a unilateral *tbc1d32* knockdown mainly limited to neural tissues (Supplemental Figure 4). A high dose (20 ng) of either Mo1 or Mo2 led to severe developmental defects, including curvature of the body axis and microphthalmia/anophthalmia of the injected embryos (data not shown). No discernable eye malformations were observed upon injection of a control Mo.

**Figure 4:**
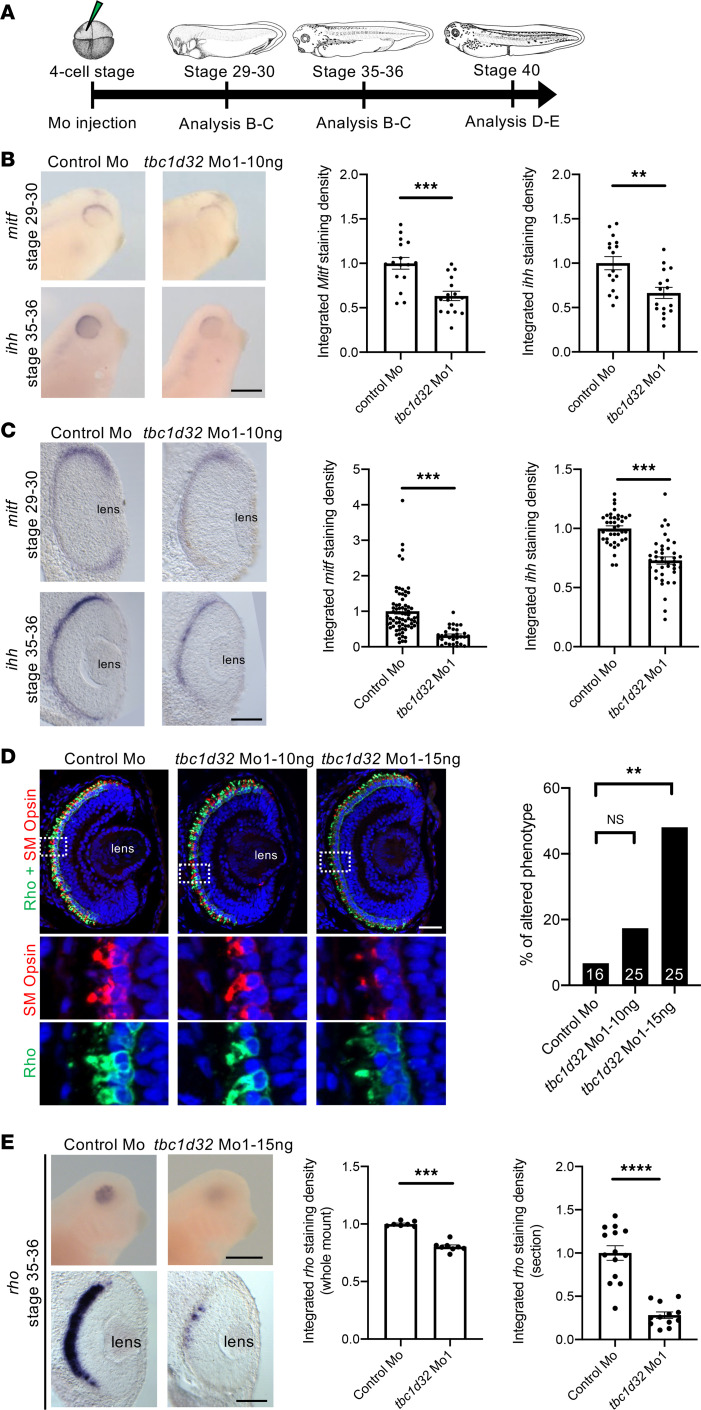
*Xenopus* RPE and photoreceptor marker expression following *tbc1d32* knockdown. (**A**) Diagram of the experimental design. Whole mount (**B**) or retinal sections (**C**) following in situ hybridization against *mitf* or *ihh*, respectively, on embryos injected with control Mo or *tbc1d32* Mo1. Scatterplots represent the quantification of the integrated density of the staining per eye relative to control Mo; each dot corresponds to 1 eye or 1 section, respectively. (**D**) Rho and SM opsin immunolabeling on retinal sections of embryos injected with control Mo or embryos injected with 2 doses of *tbc1d32* Mo1 (10 or 15 ng). Lower panels, enlargement of the areas indicated by white dashed boxes in the upper panels. The bar plot represents the proportion of eyes with altered staining of Rho and SM opsin for each condition. The number of eyes analyzed per condition is indicated in each bar. Rho, rhodopsin; SM opsin, short and middle wavelength cone opsin. (**E**) Upper panels, whole-mount in situ hybridization against *rhodopsin* in embryos injected with control Mo or *tbc1d32* Mo1. Lower panels, transverse retinal sections of control and morphant embryos. The scatterplots represent the quantification of the integrated density of *rhodopsin* staining relative to control Mo; each dot corresponds to 1 eye (left) or 1 section (right). For all scatterplots, data are represented as mean ± SEM. **P < 0.01; ***P < 0.001; ****P < 0.0001; Fisher’s exact test (**D**); 2-tailed Mann-Whitney test (**B**, **C**, and **E**). Scale bars = 400 μm for whole-mount embryos and 40 μm for sections.

As we suspected hypomorphic pathogenicity in *TBC1D32* patients, we tested lower doses of *tbc1d32* Mo. At a dose of 10 or 15 ng of Mo1, *Xenopus* embryos no longer displayed a microphthalmic phenotype, and we did not detect retinal toxicity using a cell death assay (Supplemental Figure 5). By contrast, morphant embryos exhibited retinal pigmentation defects, comprising decreased intensity and discontinuity in pigmentation on retinal sections compared with controls (Figure 3C). This phenotype was similar upon Mo2 injection (Supplemental Figure 6A) and was partially restored upon coinjection with Mo-resistant *tbc1d32* mRNAs (i.e., not recognized by the Mo), demonstrating its specificity (Figure 3C). We further analyzed actin cytoskeleton organization in RPE cells by phalloidin staining of dissected eyes injected with Mo1 or Mo2 (Figure 3D and Supplemental Figure 6B). In controls, phalloidin staining was uniform, outlining cell membranes and highlighting the typical hexagonal shape of RPE cells, whereas the staining was heterogeneous and irregular in *tbc1d32* morphants, highlighting an altered shape of RPE cells. We hence investigated the impact of *tbc1d32* knockdown on RPE differentiation using microphthalmia-associated transcription factor (*mitf*) and *ihh* as RPE markers (14). Using in situ hybridization, we found that their expression was significantly decreased in the retinas of embryos injected with Mo1 or Mo2 compared with controls (Figure 4, A–C, and Supplemental Figure 7, A and B). This decrease was verified by qPCR (Supplemental Figure 8). Importantly, this effect was partially rescued upon coinjection with Mo-resistant *tbc1d32* mRNAs (Supplemental Figure 9). Collectively, these data suggest a key role for *tbc1d32* in RPE differentiation.

We also explored whether photoreceptor development was affected upon *tbc1d32* knockdown. By immunofluorescence (IF) at stage 41, when photoreceptors are well differentiated, we found that the vast majority of morphant retinas injected with 10 ng of Mo1 exhibited normal labeling in the ONL of both rhodopsin and SM cone opsin (Figure 4D). By contrast, at a higher dose of 15 ng, around half of the morphant embryos showed reduced staining. To determine whether these defects arise early during photoreceptor differentiation, we assessed *rhodopsin* expression at stage 35–36, when *rhodopsin* RNA begins to be detected. We found significantly reduced staining in *tbc1d32* morphant retinas compared with controls (Figure 4E), which was verified following Mo2 injections (Supplemental Figure 7C). Taken together, *tbc1d32* knockdown results in RPE defects, while photoreceptors are affected in a dose-dependent manner, being either largely preserved or exhibiting obvious differentiation defects.

### Xenopus RPE ciliogenesis is affected upon tbc1d32 knockdown.

**Figure 5:**
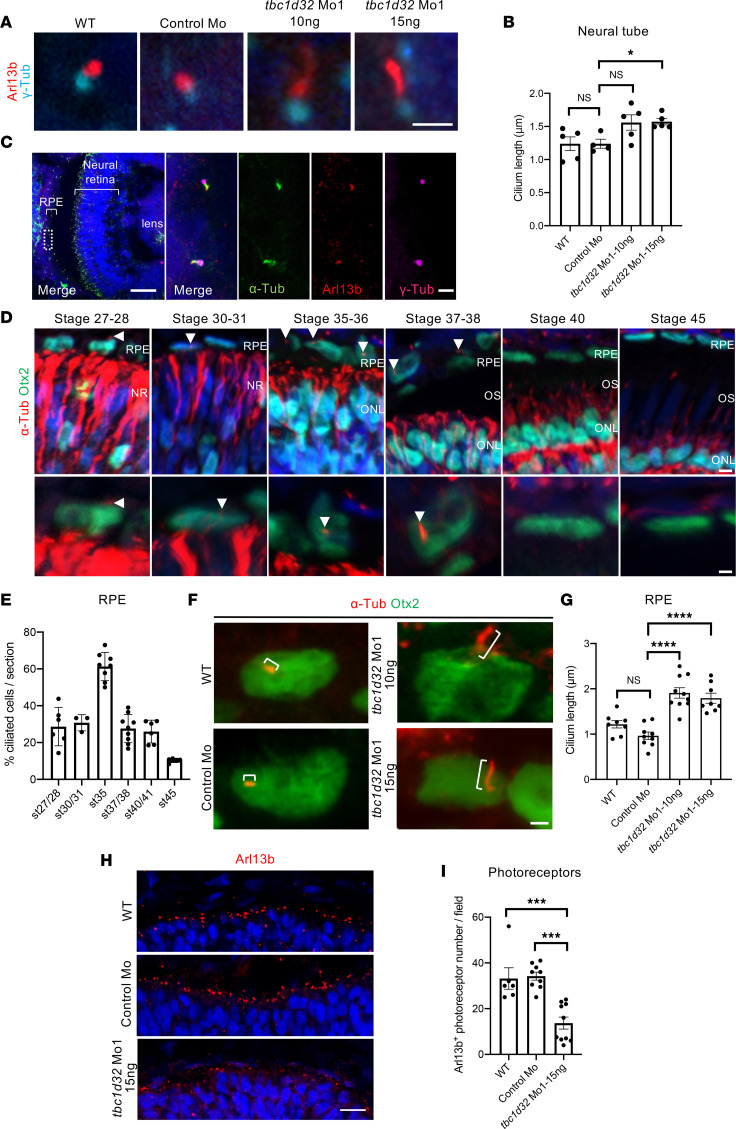
*Xenopus* ciliogenesis following *tbc1d32* knockdown. (**A**) Arl13b and γ-Tubulin (γ-Tub) immunolabeling on neural tube sections of stage 35–36 wild-type (WT), control Mo, or *tbc1d32* Mo1 embryos. (**B**) Scatterplot shows the mean length of neural tube cilia. Each dot corresponds to the mean length for 1 embryo. (**C**) Immunolabeling of cilia markers, acetylated α-Tubulin (α-Tub), Arl13b, and γ-Tub, on retinal sections of stage 35–36 WT embryo. The right panels show an enlargement of the area delineated by a white dotted box in the left panel. (**D**) α-Tub immunolabeling of RPE cilia (arrowheads) at different stages. Sections are costained with Otx2 to identify RPE cells, which are enlarged in the lower panels. NR, neural retina; ONL, outer nuclear layer; OS, outer segment. (**E**) Proportion of ciliated cells among RPE cells at different stages (st). (**F**) α-Tub and Otx2 immunolabeling showing primary RPE cilia (brackets) on retinal sections of stage 35 WT, control Mo, or *tbc1d32* Mo1 embryos. (**G**) Scatterplot with bars showing the mean cilia length in RPE cells. Each dot corresponds to the mean length for 1 embryo. (**H**) Immunolabeling of Arl13b showing the photoreceptor connective cilium on retinal sections of stage 35 WT, control Mo, or *tbc1d32* Mo1 embryos. (**I**) The scatterplot shows the mean number of Arl13^+^ ciliated photoreceptors in 1 field of the central retina. Each dot corresponds to 1 embryo. All data are represented as mean ± SEM. *P < 0.05, ***P < 0.001, ****P < 0.0001; 2-tailed Mann-Whitney test. Scale bars = 2 μm in **A** and **F**, 50 μm and 2 μm for enlargements in **C**, 5 μm and 2 μm for enlargements in **D**, 25 μm in **H**.

Defects in primary cilia morphology, namely curled cilia, were reported in both neural progenitor cells of *Tbc1d32^–/–^* mouse embryos and in renal tubules of zebrafish *tbc1d32* morphants (10). Consistently, by staining with the widely used cilium markers Arl13b (ciliary shaft marker) and γ-Tubulin (γ-Tub; basal body marker), we detected ciliogenesis defects in the neural tube of *Xenopus* *tbc1d32* morphants, which displayed longer primary cilia than controls (Figure 5, A and B). We thus assayed if the RPE developmental defects in the *tbc1d32* morphants were also associated with ciliogenesis defects. As primary cilia are necessary for maturation of mouse RPE but disappear in fully differentiated cells (15), we first characterized the developmental window during *Xenopus* retinogenesis when RPE cilia were present. We used the RPE marker Otx2, to delineate cells, and acetylated α-Tubulin and γ-Tub, to label primary cilia. First, we verified that acetylated α-Tubulin colocalized with Arl13b along the cilium and with γ-Tub at the basal body level, verifying that it is a reliable marker of the *Xenopus* RPE cilium (Figure 5C). We detected ciliated RPE cells in the presumptive retina from stage 27–28. The percentage of ciliated cells increased and peaked at stage 35, then decreased from stage 37–38 onward (Figure 5, D and E). In *tbc1d32* morphant retinas of stage 35 embryos, the mean cilia length was significantly higher than in control retinas (Figure 5, F and G, and Supplemental Figure 10, A and B). We then analyzed photoreceptor ciliogenesis and found a significant decrease in the number of photoreceptors showing Arl13b staining in *tbc1d32* morphant retinas compared with controls (Figure 5, H and I, and Supplemental Figure 10, C and D). In conclusion, *tbc1d32* knockdown impacted ciliogenesis in both *Xenopus* RPE and photoreceptor cells, albeit in different ways.

### Human TBC1D32 fibroblasts display a primary cilium defect.

Human fibroblasts have long constituted a classical screening assay for ciliary defects (16). Therefore, we cultured fibroblasts from a skin biopsy of patient 1 to better study the expression of both mutant *TBC1D32* alleles and their impact on ciliogenesis. Using primers specific to exons 1 and 8 to assay the c.317+5G>A allele, we detected a 931 bp band in control cells and 3 bands in the patient cells (Supplemental Figure 11A): the first was similar in size to the control band but contained the last 18 bp of intron 2 (Supplemental Figure 11B) that introduced a PTC; the second and most predominant was 769 bp and corresponded to the in-frame skipping of exon 2 (Supplemental Figure 11C), as observed in patient WBCs; the third was 591 bp and corresponded to the skipping of exons 2 and 3 that introduced a PTC (Supplemental Figure 11D). Thus, only transcript 2 was predicted to lead to translation, which corroborated our observations in WBCs, despite the less comprehensive analysis in this cell type. Using primers specific to exons 6 and 11 to assay the c.846delTCCTA allele, we detected an approximately 600 bp amplicon in control and patient cells (Supplemental Figure 11E), the latter of which contained a transcript with the 5 bp deletion in exon 7 (Supplemental Figure 11F) that introduced a PTC. These results suggested the mutant transcript did not undergo nonsense-mediated decay, which was consistent with the qPCR results showing similar levels of *TBC1D32* expression in control and patient fibroblasts (Supplemental Figure 11G).

**Figure 6:**
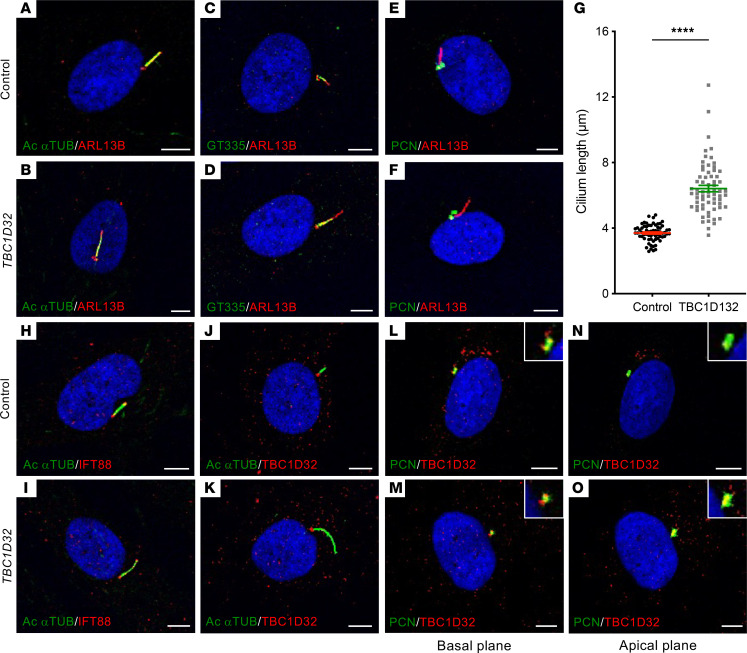
Morphology of the primary cilium in human fibroblasts. IF studies and single plane confocal imaging to assay ciliogenesis in control (**A**, **C**, and **E**) and *TBC1D32* (**B**, **D**, and **F**) fibroblasts with antibodies directed against ARL13B and acetylated α-tubulin (Ac αTUB; **A** and **B**), GT335 (**C** and **D**), and PCN (**E** and **F**). (**G**) Quantification of cilium length in control and *TBC1D32* fibroblasts. Data are represented as mean ± SEM; *n* = 66 cells; ****P < 0.0001; Student’s 2-tailed *t* test. IF studies to assay intraflagellar transport in control (**H**) and *TBC1D32* (**I**) fibroblasts with antibodies directed against IFT88 and Ac αTUB. IF studies in control (**J**) and *TBC1D32* (**K**) fibroblasts to assay the localization of TBC1D32 in relation to Ac αTUB. IF studies in control (**L** and **N**) and *TBC1D32* (**M** and **O**) fibroblasts to assay the localization of TBC1D32 in relation to PCN in a basal (**L** and **M**) and apical (**N** and **O**) plane. Insets show a 2-fold magnification of the labeled basal bodies/centrosomes in each panel. Scale bars = 5 μm.

We assayed ciliogenesis by IF studies of ARL13B with the axoneme markers acetylated α-tubulin and polyglutamylated tubulin (GT335) and the basal body marker pericentrin (PCN), which is present in both the mother and daughter centrosomes (17). We detected a similar spatial distribution between control and *TBC1D32* fibroblasts (Figure 6, A–F). By contrast, and consistent with the *Xenopus* data, we detected a significantly elongated ARL13B-labeled cilium in the patient cells compared with controls (Figure 6, A–G). We also assayed intraflagellar transport (IFT) by IF studies of IFT88 (18, 19), which correctly extended beyond acetylated α-tubulin at both ends of the cilium in control and patient cells (Figure 6, H and I). Last, consistent with the qPCR data, we clearly detected TBC1D32 using an antibody specific to an epitope encoded by exons 24 to 28. TBC1D32 was located basal to acetylated α-tubulin in control and patient fibroblasts (Figure 6, J and K), where it overlapped with PCN (Figure 6, L–O). Taken together, TBC1D32 localized to the centrosomes of the primary cilium in human fibroblasts, and mutant TBC1D32 proteins led to elongation defects.

### TBC1D32 iPSC-derived RPE has an elongated cilium and disrupted tight junctions.

To unravel the pathophysiology associated with *TBC1D32* mutations, we reprogrammed the fibroblasts of patient 1 into iPSCs to subsequently generate highly relevant human retinal models. Two *TBC1D32* iPSC lines were confirmed as pluripotent by the expression of the host pluripotency markers (Supplemental Figure 12, A and B) and germline markers following an embryoid body (EB) differentiation assay (Supplemental Figure 12C). In addition, we confirmed genomic stability by testing the copy number of the recombination hotspots in iPSCs (20) (Supplemental Figure 12D). Interestingly, contrasting the fibroblast data, *TBC1D32* expression levels in the 2 patient iPSC lines were lower than in control cells (Supplemental Figure 12E). Last, we verified the presence of the causative *TBC1D32* mutations c.317+5G>A in exon 2 and c.846delTCCTA in exon 7 in the patient iPSC lines by Sanger sequencing (Supplemental Figure 12F).

**Figure 7:**
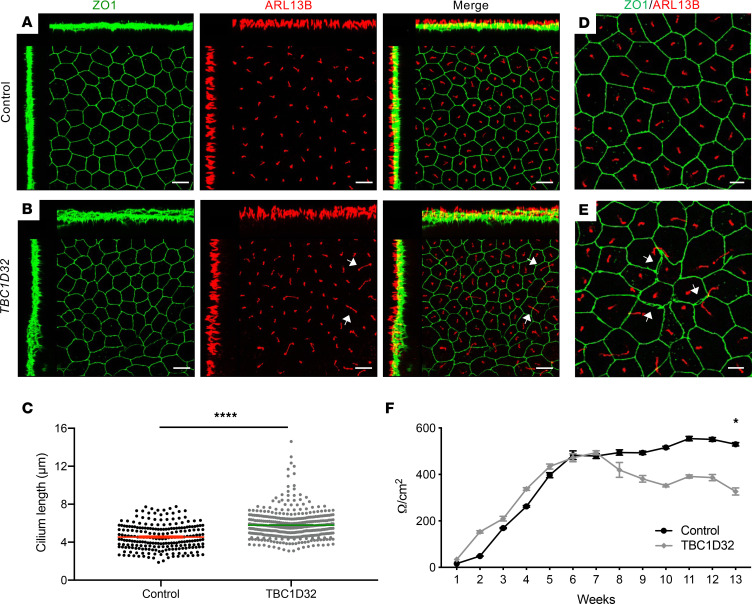
Morphology of the human iPSC-derived RPE. IF studies and maximum intensity projection (MIP) confocal imaging of control (**A**) and *TBC1D32* (**B**) iPSC-derived RPE using antibodies directed against ZO1 and ARL13B. White arrows indicate elongated cilia. The disrupted *TBC1D32* RPE monolayer can be seen on the orthogonal planes as compared with control. Scale bars = 10 μm. (**C**) Quantification of the cilium length in control (*n* = 229 cells) and *TBC1D32* iPSC-derived RPE (*n* = 416 cells). Data are represented as mean ± SEM; ****P < 0.0001; Student’s 2-tailed *t* test. (**D**) Higher magnification of control iPSC-derived RPE showing the regular cobblestone morphology and cilia length. (**E**) Higher magnification of *TBC1D32* RPE showing irregularly shaped cells, separated tight junctions (white arrows), and ZO1 aggregates. Scale bars = 5 μm. (**F**) Weekly TER measurements expressed in Ω/cm^2^ in control (black line) and *TBC1D32* (gray line) iPSC-derived RPE. Data are represented as mean ± SEM; *P < 0.05; *n* = 4 inserts; 2-tailed Mann-Whitney test.

We then differentiated the patient and control lines into iPSC-derived RPE. Due to the different qPCR profiles observed between fibroblasts and iPSCs, we assayed *TBC1D32* expression in the iPSC-derived RPE and detected lower levels in the patient RPE compared with controls (Supplemental Figure 13A), further suggesting tissue-specific regulation. By IF analysis, TBC1D32 partially colocalized with PCN in control and patient iPSC-derived RPE, as in primary fibroblasts, but the staining intensity was lower in the patient RPE (Supplemental Figure 13, B and C), consistent with the qPCR results. We then assayed the morphology of the *TBC1D32* iPSC-derived RPE from 12 weeks postseeding. Colabeling of the apical tight junction marker ZO1 and ARL13B demonstrated a regular cobblestone morphology and primary cilium staining for the control RPE (Figure 7A). By contrast, the morphology of the *TBC1D32* patient iPSC-derived RPE was highly irregular, with cilia significantly longer than controls (Figure 7, B and C). Furthermore, we detected gaps between cells, and ZO1 aggregates at the contact points of multiple cells, in the *TBC1D32* RPE that were absent from control RPE (Figure 7, D and E). The disrupted tight junctions were verified by trans-epithelial resistance (TER) measurements, which steadily decreased in the *TBC1D32* RPE from 6 weeks postseeding to levels significantly lower than control RPE at 13 weeks (Figure 7F). In conclusion, the *TBC1D32* patient iPSC-derived RPE presented with a primary cilium defect and disrupted apical tight junctions.

### TBC1D32 iPSC-derived RPE displays an epithelial-mesenchymal transition–like phenotype.

**Figure 8:**
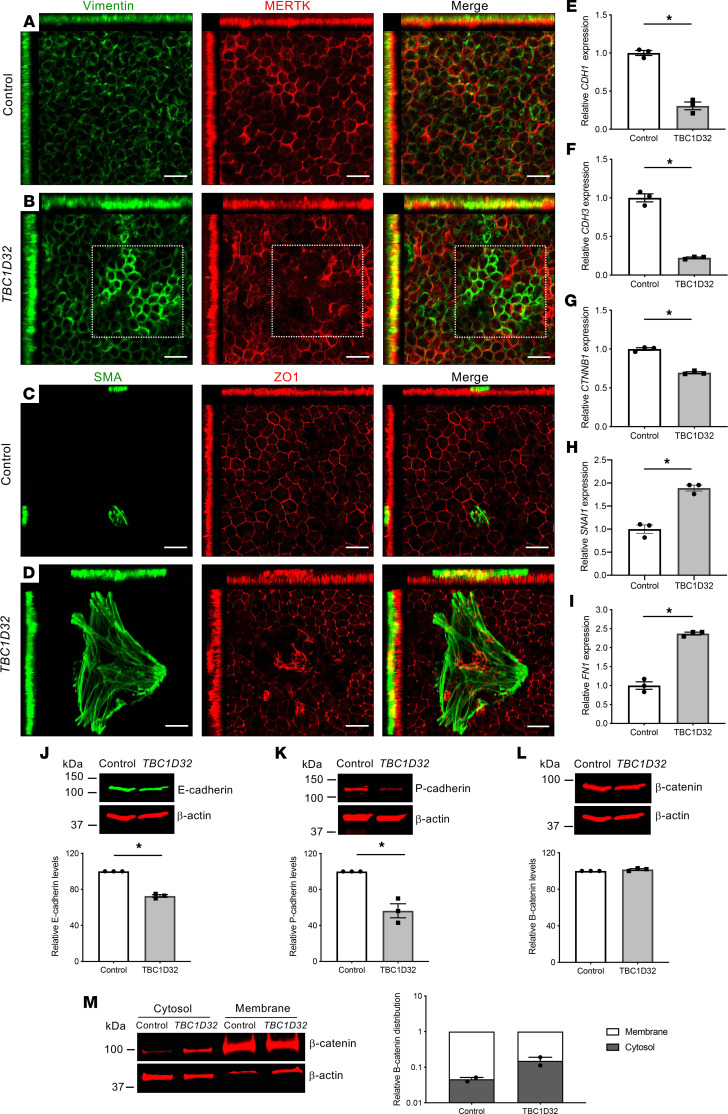
EMT-like phenotype in human *TBC1D32* iPSC-derived RPE. IF studies and MIP confocal imaging of control (**A**) and *TBC1D32* (**B**) iPSC-derived RPE using antibodies directed against vimentin and MERTK. The boxed area in **B** indicates areas of upregulated vimentin and disrupted MERTK expression. Scale bars = 20 µm. IF studies and MIP confocal imaging of control (**C**) and *TBC1D32* (**D**) iPSC-derived RPE using antibodies directed against SMA and ZO1. Scale bars = 20 μm. qPCR analysis of *CDH1* (**E**), *CDH3* (**F**), *CTNNB1* (**G**), *SNAI1* (**H**), and *FN1* (**I**) in control and *TBC1D32* RPE. Data are represented as mean ± SEM; *n* = 3 technical replicates; *P < 0.05; 2-tailed Mann-Whitney test. Representative Western blot analysis of E-cadherin (**J**), P-cadherin (**K**), and β-catenin (**L**) expression and quantification relative to the β-actin loading control; the same membrane was hybridized with 2 different primary antibodies in panels **J** and **L**. Data represented as mean ± SEM; *n* = 3 blots; *P < 0.05; 2-tailed Mann-Whitney test. (**M**) Representative Western blot analysis and quantification of 2 independent blots of β-catenin distribution in the cytosol versus membrane fractions of control and *TBC1D32* iPSC-derived RPE relative to the β-actin loading control and displayed on a logarithmic scale. Data are represented as mean ± SEM.

Primary cilium defects in RPE reportedly provoke an epithelial-mesenchymal transition–like (EMT-like) phenotype, characterized initially by a loss of tight junctions (21). We thus assayed the *TBC1D32* RPE for signs of cytoskeletal changes associated with EMT by testing the expression of vimentin and smooth muscle actin (SMA) (22) (Figure 8, A–D). A low and relatively regular intensity of vimentin expression was detected in the control iPSC-derived RPE, whereas islands of upregulated expression were detected in the patient RPE, which were associated with a loss of MERTK expression, further validating the loss of RPE identity. Similarly, large and abundant SMA-positive islands were detected in the *TBC1D32* iPSC-derived RPE, compared with controls, which were associated with disrupted ZO1 expression (Supplemental Figure 13, D–F). Thus, the disrupted apical junctions in the *TBC1D32* iPSC-derived RPE were accompanied by a transition to a mesenchymal state.

These observations were further validated by gene expression levels of other EMT markers (Figure 8, E–I). The genes encoding the adherens junction markers E-cadherin (*CDH1*), P-cadherin (*CDH3*), and β-catenin (*CTNNB1*) showed reduced expression levels in the *TBC1D32* RPE, compared with controls, validating the loss of epithelial cell-cell contacts. Furthermore, the transcription factor SNAIL (*SNAI1*), which mediates EMT, and fibronectin 1 (*FN1*), a marker of EMT, showed increased expression levels. Consistently, Western blot analysis of E- and P-cadherin showed significantly decreased levels in the patient RPE compared with controls (Figure 8, J and K). In contrast to the qPCR data, β-catenin showed similar levels in control and patient RPE (Figure 8L). We thus analyzed its subcellular distribution and showed that it was predominately localized to the membrane fraction in control RPE and to the cytosolic fraction in the patient RPE (Figure 8M). This difference was consistent with the release of β-catenin from the membrane, which allows it to act as a transcriptional activator of EMT genes, such as *SNAI1* (23). Taken together, the *TBC1D32* iPSC-derived RPE displays altered morphology and deregulated expression profiles that are hallmarks of EMT activation.

### TBC1D32 iPSC-derived RPE shows altered functionality.

**Figure 9:**
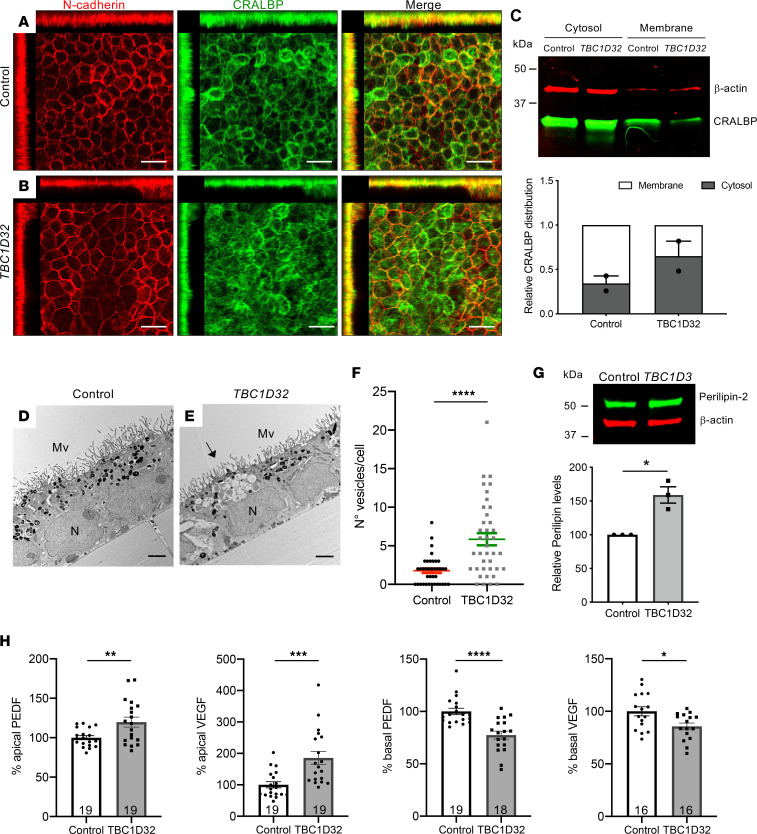
Retinosome accumulation and altered secretion in human *TBC1D32* iPSC-derived RPE. IF studies and MIP confocal imaging of control (**A**) and *TBC1D32* (**B**) iPSC-derived RPE using antibodies directed against N-cadherin and CRALBP. Redistribution of N-cadherin and CRALBP can be seen on the orthogonal planes. Scale bars = 10 μm. (**C**) Representative Western blot analysis and quantification of 2 independent blots of CRALBP distribution in the cytosol versus membrane fractions of control and *TBC1D32* iPSC-derived RPE relative to the β-actin loading control. Data represented as mean ± SEM. TEM of control (**D**) and *TBC1D32* (**E**) iPSC-derived RPE showing apical microvilli (MV), basal nuclei (N), pigmented melanosomes, and vesicles corresponding to lipid droplets (black arrow). Scale bar = 2 μm. (**F**) Quantification of the number of vesicles per cell in control (*n* = 38 cells) and *TBC1D32* (*n* = 39 cells) RPE. Data are represented as mean ± SEM; ****P < 0.0001; Student’s 2-tailed *t* test. (**G**) Representative Western blot analysis of perilipin expression and quantification relative to the β-actin loading control. Data represented as mean ± SEM. *n* = 3 blots; *P < 0.05; 2-tailed Mann-Whitney test. (**H**) ELISA of PEDF and VEGF secretion in apical and basal chambers of control and TBC1D32 RPE cultured on Transwell membrane inserts. Data are expressed as a percentage of control; *P < 0.05; **P < 0.01; ***P < 0.001; ****P < 0.0001; the number of samples are indicated within bars. Student’s 2-tailed *t* test.

The loss of tight and adherens junctions reportedly affects the apical-basal polarity of RPE with an impact on functionality (21). Hence, we assayed the expression of CRALBP, an RPE marker and actor of the visual cycle, together with N-cadherin, an adherens junction and EMT marker (22) (Figure 9, A and B). We detected a regular membrane staining of N-cadherin in control RPE compared with notable patches of discontinuous N-cadherin staining in the patient RPE. Similarly, we detected reduced CRALBP staining at the membrane and an increased signal in the cytosol in the patient RPE compared with controls. This shift was verified by Western blot analyses (Figure 9C). Furthermore, the most pronounced areas of CRALBP staining showed reduced N-cadherin levels. Moreover, we detected a decrease in expression of the corresponding gene, *RLBP1*, as well as other visual cycle genes, *LRAT* and *RPE65* in *TBC1D32* RPE compared with controls (Supplemental Figure 13, G–I). *ABCA4*, which is involved in the visual cycle in photoreceptors, was recently described to be also expressed in the RPE (24), and accordingly, its expression was also reduced in the patient RPE (Supplemental Figure 13J).

To determine whether the differential expression of these visual cycle actors in the patient RPE impacted its function, we assayed for the accumulation of retinoids, detectable as lipid droplets termed retinosomes (25) by transmission electron microscopy (TEM). We detected an accumulation of lipid droplets in the *TBCD132* RPE as compared with controls (Figure 9, D–F), which were identified as retinosomes by the specific marker perilipin-2 (Figure 9G). The significant abundance of retinosomes prompted us to assay the polarized secretion of the angiogenic factors pigment epithelium-derived factor (PEDF) and VEGF, another key RPE function, by ELISA (26). We detected a significant increase in apical, and a significant decrease in basal, PEDF and VEGF secretion in the *TBC1D32* RPE, as compared with controls (Figure 9H). Taken together, the cellular changes in the *TBC1D32* RPE result in disrupted retinoid cycling and altered secretion balance.

### TBC1D32 iPSC-derived retinal organoids show defective trafficking to outer segments.

**Figure 10:**
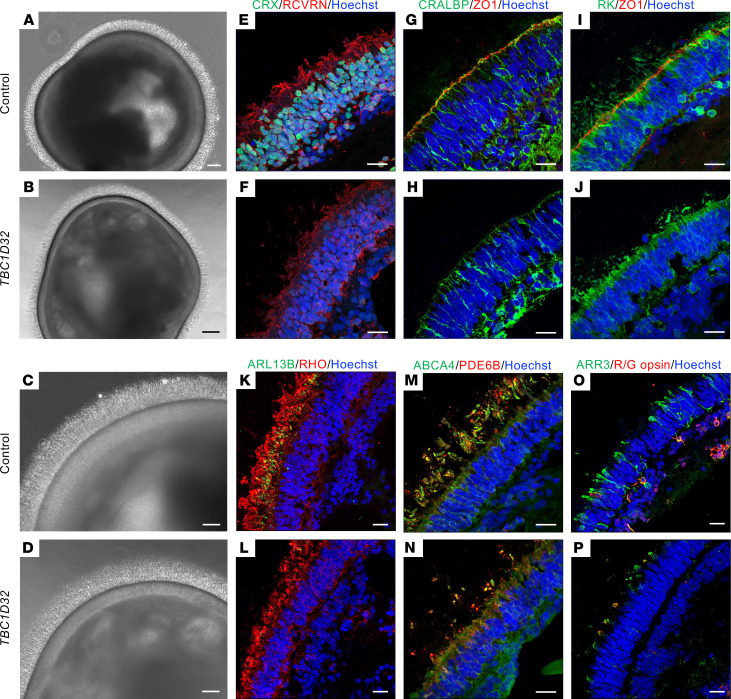
Morphology of human iPSC-derived retinal organoids. Brightfield microscopy of control (**A**) and *TBC1D32* (**B**) retinal organoids at day 225 of differentiation showing the brush border. Scale bars = 100 μm. Higher magnification of the ONL of control (**C**) and *TBC1D32* (**D**) organoids. Scale bars = 50 μm. IF studies and MIP confocal imaging of control (**E**, **G**, and **I**) and *TBC1D32* (**F**, **H**, and **J**) organoids with antibodies directed to CRX and RCVRN (**E** and **F**) to assay general photoreceptor morphology, CRALBP and ZO1 to assay the OLM (**G** and **H**), and RK and ZO1 to assay the prolongation of the IS and OS (**I** and **J**). IF studies of control (**K**, **M**, and **O**) and *TBC1D32* (**L**, **N**, and **P**) organoids with antibodies directed to ARL13B and RHO (**K** and **L**) to assay the CC and prolongation of the OS, ABCA4, and PDE6B (**M** and **N**) to assay the OS of rods and/or cones, and ARR3 and R/G opsin (**O** and **P**) to assay the cones. All nuclei are labeled with Hoechst. Scale bars = 20 μm.

As photoreceptors possess a specialized cilium, the CC, we assayed whether *TBC1D32* defects also led to photoreceptor anomalies in iPSC-derived retinal organoids (27). Brightfield imaging showed that control and *TBC1D32* organoids had a similar morphology with a brush border (Figure 10, A and B) containing IS and OS-like structures and a distinct lamina corresponding to the ONL (Figure 10, C and D). This organization was confirmed by IF studies of CRX and recoverin (RCVRN), although staining was visibly reduced in the patient organoids (Figure 10, E and F). Furthermore, we assayed the expression of ZO1 and CRALBP, markers of the outer limiting membrane (OLM) formed by the end feet of Müller glial cells. The OLM was clearly discernible by the colocalized CRALBP/ZO1 signals in the control organoids, whereas we detected only a faint CRALBP signal and no ZO1 signal in the *TBC1D32* organoids (Figure 10, G and H). Moreover, costaining of ZO1 and rhodopsin kinase (RK) showed that RK expression extended beyond the OLM into the IS/OS layer in control organoids but not as far in the *TBC1D32* organoids (Figure 10, I and J). We thus investigated the CC by assaying the expression of ARL13B in parallel to rhodopsin (RHO). In control organoids, ARL13B was visible as punctate dots close to the ONL with RHO extending beyond into the OS, whereas in *TBC1D32* organoids, ARL13B was only faintly discernible and RHO remained close to the ONL (Figure 10, K and L). The RK and RHO expression profiles suggested that trafficking to the OS was impaired; thus, we tested the expression of the cone and rod OS marker ABCA4 and the rod-specific OS marker PDE6B. ABCA4 and PDE6B staining was clearly detectable in the OS of the control organoids but visibly reduced in the *TBC1D32* organoids (Figure 10, M and N). In addition, arrestin 3–positive (ARR3-positive) cones of varying lengths were identified in the control organoids, a subset of which coexpressed the more mature red/green (R/G) opsin cone marker (Figure 10O). By comparison, the cones in the *TBC1D32* organoids appeared stunted with a distinctly visible colocalization signal at the edge of the cells beyond the ONL (Figure 10P). In conclusion, in addition to affecting the RPE, *TBC1D32* variants directly affect photoreceptors at the level of the CC and result in impaired trafficking to the OS in both cones and rods.

## Discussion

With the technological advancements of next-generation sequencing, the genetic diagnosis yield of IRDs has considerably increased over the last 10 years (28). However, recently, the slope of the identification curve has reached a plateau (RetNet, https://sph.uth.edu/retnet), raising challenges for genetic counseling and therapeutic opportunities for patients. Furthermore, the genetic landscape for IRDs is complexifying, as it becomes increasingly evident that mutations in the same gene can give rise to differential phenotypes or even clinically distinct disorders (29). *TBC1D32* is now another perfect example of the breakdown in borders between disorders. To date, *TBC1D32* variants were only associated with OFD syndrome, a severe malformative disorder. However, here, we report pathogenic *TBC1D32* variants in 4 patients from 3 independent families presenting clinically with RP. Furthermore, by combining an in vivo *Xenopus* model and human iPSC-derived retinal models, we describe a critical role for *TBC1D32* in retinal differentiation and ciliogenesis and demonstrate that reduced *TBC1D32* levels impact the RPE and photoreceptors. We thus identify *TBC1D32* as a causative IRD gene.

OFD syndromes form a rare subgroup of ciliopathies characterized by oral cavity, facial and digital anomalies with or without heart defects, polycystic kidney disease, or corpus callosum agenesis (30). There is a high clinical heterogeneity linked to 16 causative genes. The most frequent is the X-linked *OFD1*, which encodes a centrosomal protein. Interestingly, rare *OFD1* patients have been described as presenting with nonsyndromic retinal degeneration (31), suggesting that variants in other *OFD* genes may also give rise to IRDs. In 2014, loss-of-function *TBC1D32* variants were first described as associated with the OFD syndrome type IX (11). Clinical symptoms ranged from midline cleft, anophthalmia, polydactyly to choanal atresia, agenesis of corpus callosum, vermis and pituitary hypoplasia, and hydrocephalus. Subsequently, 7 patients from 5 families were reported as carrying different pathogenic *TBC1D32* variants and presenting with syndromic ciliopathies (32). It is noteworthy that 1 patient from a Finnish family with compound heterozygous indels in *TBC1D32* also exhibited a progressive retinal dystrophy, but this was not further explored (33).

Taking into account the clinical data in relation to age, none of the patients reported here showed signs of *TBC1D32*-related OFD syndrome. It should be noted, though, that MRI revealed mild subclinical alterations, which suggests that *TBC1D32* variants may give rise to a disease spectrum with a certain threshold for the appearance of clinical signs. It is likely that type or severity of the associated variant accounts for this spectrum. We now raise the number of reported pathogenic *TBC1D32* variants, identified in 8 unrelated families, to 10: 1 missense (c.3513G>T), 2 nonsense (c.1267G>T; c.3724C>T), 3 splicing (c.317+5G>A; c.1141-1G>A; c.1372+1G>T), and 4 indels (c.18_27del; c.846delTCCT; c.1165_1166dup; c.2151del) (refs. 32–34 and this study). Although it is possible that nonsense and indel *TBC1D32* variants result in loss of function, splicing and missense variants are more ambiguous.

The c.317+5G>A splicing variant, carried by patient 1 in *trans* to a frameshift indel, only results in mild RP, which is likely explained by the resulting in-frame exon skipping that may result in a shorter partially functional protein. Similarly, the c.1141-1G>A variant, carried by patient 2 in *trans* to a frameshift indel, which promotes the creation of multiple aberrant splicing variants, also did not result in severe syndromic disease. However, this patient presented with additional renal defects, which were possibly of a ciliopathic nature. An association between altered ciliary function and the development of renal cysts has been linked to variants in the ciliary function-related genes *PKD1/PKD2* (35). Patient 2 was negative for pathogenic *PKD1/PKD2* variants, raising the possibility that the renal anomalies may be due to the *TBC1D32* variants. This is supported by the zebrafish *tbc1d32* morphant model that shows dose-dependent ciliary defects in the distal kidney tubules (10) and the association of renal anomalies with other ciliopathies (7). Along this line, a recent study described developmental cochlear defects in the knockout *Tbc1d32* mouse model (36), and we also detected *tbc1d32* in the *Xenopus* otic vesicle, suggesting that *TBC1D32* variants may also provoke ciliopathies with deafness associated. Finally, the mildest *TBC1D32*-associated phenotype reported to date is the later onset RP in patients 3 and 4 carrying the missense c.3513G>T variant in *trans* to a nonsense variant. The predicted structural changes associated with this variant suggest the production of a mutant protein, which we hypothesize may be partially functional to account for the moderate phenotype. Taken together, and in consideration of the dose-dependent phenotypic severity in the *Xenopus tbc1d32* morphants, we propose that hypomorphic *TBC1D32* variants give rise to RP and loss-of-function variants to OFD syndrome.

Pertinently, none of the patients showed ocular malformations, whereas they all presented with features of retinal degeneration that were compatible with both RPE and photoreceptor defects. These clinical data are strongly supported by our functional data. First, the phenotypes of the *Xenopus* *tbc1d32* morphants and the human *TBC1D32* iPSC-derived RPE models highlighted RPE differentiation defects that affected functionality. Most notably, the slowing down of retinoid recycling, as evidenced by retinosome accumulation, is reminiscent of the RPE defect associated with mutations in the visual cycle protein *RPE65*, which causes Leber congenital amaurosis (37). Visual cycle disruption reduces the availability of 11 *cis*-retinol, which subsequently impacts the phototransduction process causing visual impairment. Second, the *Xenopus* morphants and iPSC-derived retinal organoids showed photoreceptor differentiation anomalies and disturbed trafficking of photoreceptor proteins to the OS, which would affect their function. In addition, the disrupted apical VEGF secretion identified in the *TBC1D32* iPSC-derived RPE would also impact neurodevelopment and photoreceptor protection throughout life (38), further impacting phototransduction and vision.

The disrupted ciliogenesis of the RPE and photoreceptors in both the *Xenopus* and human models leads us to propose that *TBC1D32*-associated RP is a retinal ciliopathy (7). In particular, we show that TBC1D32 defects result in elongated primary cilia in fibroblasts, the neural tube, and RPE cells. Ciliary length is determined by an interplay between ciliary assembly and disassembly processes, which rely on an effective bidirectional IFT system (39). There have been examples in the past of gene variants associated with retinal degeneration that result in elongated cilia. For example, variants in the gene encoding IFT172 have been associated with nonsyndromic or syndromic forms of RP (40, 41), and elongated cilia were detected on patient fibroblasts (41). Similarly, variants in the gene encoding the transition zone protein CEP290 (42), associated with the early-onset retinal dystrophy Leber congenital amaurosis (LCA), resulted in elongated primary cilia on patient fibroblasts (43). Furthermore, these fibroblasts showed normal IFT88 distribution, similar to our observations on *TBC1D32* cells, indicating that anterograde IFT was normal. Finally, variants in the gene encoding NPHP5/IQCB1 also associated with LCA were recently shown to provoke elongated primary cilia in patient-derived fibroblasts and iPSC-derived RPE (44), further validating that elongated ciliary defects give rise to retinal ciliopathies.

Importantly, we show that the tight junction defects of the *TBC1D32* RPE were associated with an EMT-like phenotype both at the mRNA and protein levels. We did observe a slight discrepancy for β-catenin, as the decreased mRNA levels did not corroborate with the unchanged protein levels. The regulation of β-catenin in EMT, however, is highly complex due to the protein’s dual roles in adhesion and transcription, and the interplay between these pools has not been elucidated (45). Nonetheless, we showed that there was a release of β-catenin into the cytosol, which would increase the transcriptionally active pool consistent with EMT (23). We can thus only speculate that, at the time of analysis, the RPE was in a dynamic EMT state and that feedback regulations on mRNA or protein levels were in process. TBC1D32 is an actor of the Hedgehog (HH) pathway in primary cilia, which acts to regulate the glioma-associated oncogene (GLI) family of transcription factors (46). TBC1D32 controls the HH pathway by allowing GLI proteins to be properly localized in the primary cilia and correctly activated in response to high-level HH signaling (10). In cancer cells, it has been shown that activated GLI proteins bind and transcribe genes that control EMT (namely *SNAIL*) among other cell processes (47). Therefore, our data suggest that ciliary elongation in the RPE due to TBC1D32 disruption may cause HH signaling perturbations, which would activate EMT, causing a loss of identity and function. Although beyond the scope of this manuscript, future studies should be aimed at precisely deciphering the link between TBC1D32, HH signaling, and EMT in the retina. Last, a similar link between primary ciliary defects and EMT in the RPE was also made in a murine model of the syndromic retinal ciliopathy Bardet-Biedl syndrome (*BBS8^–/–^*), where an EMT-like phenotype was associated with defective cellular polarization and morphology (21), further validating our observations.

In general, the proteins encoded by OFD genes have been attributed to 3 functional modules in primary cilia: centriole elongation, transition zone, and intraflagellar transport (30). TBC1D32 was attributed to the elongation module as zebrafish tbc1d32 interacts with cell cycle–related kinase to regulate ciliary membrane and axonemal growth in the neural tube (10), and murine Tbc1d32 acts redundantly with the centrosomal protein Dzip1l to regulate ciliogenesis (48). We now demonstrate that TBC1D32 also regulates ciliogenesis in the retina in human and *Xenopus* models. Consistent with the fact that TBC1D32 is not associated with the intraflagellar transport module, we did not detect abnormal IFT88 distribution in *TBC1D32* fibroblasts. Interestingly though, IFT88 pooling was reported in the fibroblasts of a *TBC1D32*-associated OFD type IX patient (19) and in a *TBC1D32*-knockout hTERT-RPE1 cell line (49). Furthermore, murine *tbc1d32^–/–^* and zebrafish morphant models displayed curled axonemes enveloped by dilated ciliary membranes (10), in contrast with the elongated ciliary defect we report in the *Xenopus*-knockdown model and in *TBC1D32* patient cells. These differences could be due to a dose-dependent effect related to the amount of remaining functional TBC1D32 protein. Thus, taken together, we propose that the severity of TBC1D32 disruption will lead to phenotypic variability at the level of the primary cilium and an ensuing disease spectrum.

In conclusion, we identify *TBC1D32* as an IRD gene responsible for RP and broaden the clinical spectrum associated with *TBC1D32* variants. As we identified pathogenic variants in multiple unrelated families, and as one of the probands also presented with renal anomalies, we highly recommend that *TBC1D32* be added to the list of candidate genes to be screened for both isolated and syndromic RP.

## Methods

### Clinical investigations.

Patients underwent a comprehensive ophthalmological examination (best-corrected visual acuity, kinetic visual field, SD-OCT, SW-FAF, color fundus photography, and full-field electroretinography performed in accordance with the guidelines of the International Society for Electrophysiology of Vision). Age of onset, initial symptoms, and extraocular manifestations were also reviewed. The systemic checkup included a cerebral MRI, hypothalamic-pituitary hormone dosages, skeletal investigations, and ear, nose, throat, heart, and kidney evaluations.

### WES and bioinformatic analyses.

Targeted exome sequencing library preparation, exome capture, sequencing, and data analysis for patient 1 and his unaffected parents were performed by IntegraGen SA using Sureselect All Exon V5 (Agilent Technologies) on an Illumina HiSeq 4000, as previously described (50). WES for patient 2 was performed using the Twist Comprehensive Exome Panel (Twist bioscience) and sequenced on a HiSeq 4000 instrument with an average coverage of 100–120× at each nucleotide position. WES for patients 3 and 4 and a healthy sibling was conducted at CNAG-CRG (Centro Nacional de Análisis Genómico, Barcelona, Spain) using Sureselect All Exon V5 (Agilent Technologies), and captured libraries were paired-end (2 × 100 bp) sequenced on Illumina HiSeq 2000 platform. For patients 2 to 4, Burrows-Wheeler Aligner (V0.7.17) was used for mapping raw reads to the human genome reference sequence (build hg19), and the bioinformatic analysis was performed as described (51). For patients 3 and 4, the raw genomic data were processed in parallel through the RD-Connect analysis pipeline in the centralized RD-Connect database (52).

### Xenopus embryo collection, RNA extraction, and cloning.

*Xenopus laevis* embryos were supplied and hosted by TEFOR, Paris-Saclay’s zootechnics service. Embryos were obtained by conventional methods of hormone-induced egg laying and in vitro fertilization, staged according to Nieuwkoop and Faber’s table of development (13). Total RNA from wild-type *Xenopus* *laevis* embryos was isolated using the Nucleospin RNA XS kit (MACHEREY-NAGEL). Following reverse transcription, PCR amplification was performed using specific primers (Supplemental Table 2) designed from the *tbc1d32* sequence in the NIH National Center for Biology Information *Xenopus laevis* EST database (accession XM_041562734.1). The amplified *tbc1d32* sequence was then cloned using the ligation-free cloning system in the XhoI-linearized pCS2+ plasmid (E001, ABM) and verified by sequencing (Supplemental Figure 3).

### Xenopus whole-mount in situ hybridization.

Digoxigenin-labeled antisense RNA probes were generated according to the manufacturer’s instruction (Roche) from gifted plasmids: pBS-*ihh* (Stephen Ekker, Mayo Clinic, Rochester, Minnesota, USA); pGEM-T Easy-*mitf* (Andrea Viczian, Upstate Medical University, Syracuse, New York, USA); and pGEM-T-*rhodopsin* (Thomas Hollemann, Martin-Luther-University Halle-Wittenberg, Halle, Germany). The *tbc1d32* probe was generated from the entire *tbc1d32* coding region cloned into pCS2+. Whole-mount in situ hybridizations were carried out as described (14) and analyzed on 50 μm vibratome transverse sections.

### RNAscope in situ hybridization.

*Xenopus* embryos were fixed in 4% paraformaldehyde at 4°C for 24 hours, embedded in OCT, and sectioned at 10 μm thickness on a cryostat (Leica). The RNAscope Multiplex Fluorescent 2.0 Assay was performed according to the manufacturer’s protocols using the HybEZ oven. Opal 520 and 570 dyes were applied to slides along with DAPI counterstain.

### Morpholinos and microinjection.

Translation-blocking antisense Morpholino oligonucleotides (GeneTools) are listed in Supplemental Table 2. Five nL microinjections were performed at the 4-cell stage into 1 of the 2 blastomeres giving rise to the neural ectoderm. A total of 500 pg of *tbc1d32* mRNA (synthesized with mMessage mMachine kit, Life Technologies) and/or 10 to 20 ng of *tbc1d32* Mo1 or Mo2 were injected; a standard Mo (GeneTools) was injected as a control. For each condition, mRNA encoding GFP was coinjected as a lineage tracer. The efficacy of the 2 *tbc1d32* Mo was tested by analyzing in vivo GFP fluorescence following coinjection of a chimeric GFP construct fused downstream of the Mo-complementary sequence (Supplemental Figure 4).

### Fibroblasts and iPSCs.

Control fibroblast and iPSCs lines were previously reported (53). The skin biopsy of patient 1 was performed at the National Reference Center for Inherited Sensory Diseases (Montpellier, France). Dermal fibroblasts were reprogrammed using the CytoTune-iPS 2.0 Sendai Reprogramming Kit (Thermo Fisher Scientific), and iPSC colonies were cultured in Essential 8 Medium (Gibco) (54). Trilineage differentiation potential was determined by an EB differentiation assay as described (54). Genomic stability of iPSCs was analyzed using droplet digital qPCR analysis by Stem Genomics (Montpellier, France). iPSC genomic DNA was isolated, PCR was amplified using *TBC1D32*-specific primers (Supplemental Table 3), and the amplicons were sequenced on an Applied Biosystems 3130xL or SeqStudio genetic analyzer (54).

### iPSC-derived RPE and retinal organoids.

iPSC-derived RPE was differentiated (53) and cryostored at passage 2 (26) as described. All experiments were performed on passage 3 RPE seeded on Matrigel-coated translucent Corning cell culture inserts with high-density 0.4 μM pores (BD Biosciences). The TER of 4 independent inserts at each time point was measured using the Epithelial Volt/Ohm Meter EVOM2 (Word Precision Instruments) and expressed as an average value in Ω/cm^2^ (53). The TEM analysis of iPSC-derived RPE grown on Transwell inserts was performed as described (53). Retinal organoids were differentiated from adherent iPSCs and cultured as free-floating structures in DMEM/F12+GlutaMAX (Gibco) supplemented with taurine and retinoic acid, as described (27).

### RT-PCR and qPCR.

Total *Xenopus* RNA from at least 12 optic vesicles at stage 30, 12 whole retinas, 40 dissected RPE, or 40 neural retinas at stage 35–36 was extracted using Trizol and Nucleospin RNA Kit (MACHEREY-NAGEL). cDNA was synthesized from 290 ng of RNA with SuperScript IV (Invitrogen). For qPCR experiments, 10 ng of cDNA was amplified in triplicate using SsoFast EvaGreen Supermix (BioRad) on a C1000 thermal cycler (CFX96 real-time system, BioRad). Quantification was performed using the ΔΔCt method, and levels were normalized to *odc*, *ef1a*, and *rpl8* and expressed relative to control Mo or neural retina. Total human RNA was extracted from WBCs, fibroblasts, iPSCs, and iPSC-derived RPE using the PAXgene RNA kit (BD Biosciences), Tempus Spin RNA Isolation kit (Applied Biosystems), or RNeasy Mini Kit (QIAGEN). cDNA was synthesized from 500 ng of RNA with the SuperScript III First-Strand Synthesis System using random hexamers (Life Technologies) or from 2 μg of RNA with the High Capacity cDNA Reverse Transcription Kit (Applied Biosystems). RT-PCR amplification of patient 1’s WBCs and fibroblasts was performed using AmpliTaq Gold 360 Master Mix (Applied Biosystems) and patient 2’s WBCs using GoTaq G2 DNA Polymerase (Promega). Amplicons were either gel purified and directly sequenced (WBC patient 1) or subcloned into pGEM-T Easy vectors and then sequenced. For qPCR experiments, a 1/10 (iPSCs) or a 1/20 dilution (RPE) of the cDNA was amplified in triplicate using the FastStart SYBR Green Master mix on a LightCycler 480 II thermal cycler (Roche). Quantification was performed using the ΔΔCt method normalized to *GAPDH*, and levels were expressed relative to control. Experiments were duplicated using a second housekeeping gene. Primer sequences are presented in Supplemental Table 4.

### IF studies.

*Xenopus* embryos were fixed in 4% paraformaldehyde, and immunolabeling was performed on paraffin sections with antibodies listed in Supplemental Table 5 using standard procedures. Antigen retrieval was performed by boiling the sections in 10 mM sodium citrate and 0.05% Tween 20 for 9 minutes. For cilia analysis, sections were bleached in 10% H_2_0_2_ in PBS at 55°C. Nuclei were stained with Hoechst (MilliporeSigma). Sections were imaged with a Zeiss M2 microscope, or a Zeiss LSM 710 confocal microscope for cilia analysis, and processed with Zen software (Zeiss). For phalloidin staining, dissected eyes were fixed in 4% paraformaldehyde and labeled with Alexa Fluor 568–phalloidin (1/40, Molecular Probes). IF studies of human fibroblasts, iPSCs, EBs, RPE, and retinal organoids were performed as previously described (53–55). Primary antibodies were incubated at 4°C overnight, and secondary antibodies with 0.2 μg/mL Hoechst 33258 (MilliporeSigma) were incubated at room temperature for 1 hour (Supplemental Table 6). Samples were imaged using a Zeiss ApoTome 2 Upright wide-field microscope or confocal LSM700 microscope.

### Image quantification.

All *Xenopus* experiments were performed at least in duplicate, and results from 1 representative experiment are shown. To quantify activated caspase-3^+^–labeled cells, 6 to 9 sections per retina were analyzed using Adobe Photoshop CS4 software. In situ hybridization staining integrated density in eyes or in retinal sections was measured in the delineated region of interest using Fiji software (https://fiji.sc/). Fiji software was also used to quantify *tbc1d32* expression upon RNAscope in situ hybridization. To image and measure cilia lengths, *Z*-stacks of 11 μm retina sections were taken with 0.3 μm steps and converted into single planes by maximum projection with Fiji software. For each condition, the length of 3 to 8 cilia per retina was quantified on image stacks using the ObjectJ plugin (https://sils.fnwi.uva.nl/bcb/objectj/). The quantification of the cilium lengths in human fibroblasts and iPSC-derived RPE were performed using ImageJ and Imaris Software (Bitplane), respectively. For the RPE, 5 random regions of a Transwell filter were measured. The quantification of the SMA area was performed on a montage of 16 regions of a Transwell filter using the Imaris Software. The quantification of the lipid vesicles in the TEM images was performed manually.

### Western blot analyses.

Unless otherwise stated, hiPSC-derived RPE cells were seeded on inserts, scraped 12 weeks postseeding, and resuspended in Laemmli sample buffer containing 2-mercaptoethanol, and Western blot analysis was performed as previously described (53). The Mem-PER Plus Kit (Thermo Fisher Scientific) was used to separate the cytosolic and membrane fractions prior to mixing with Laemmli buffer. Primary and secondary antibodies (Supplemental Table 6) were incubated overnight at 4°C or 45 minutes at room temperature, respectively. Fluorescence was detected using the ODYSSEY CLx imaging system (LI-COR) and quantified with the Image Studio Lite software.

### Secretion assays.

VEGF and PEDF secretion of human iPSC-derived RPE was assayed by ELISA (R&D Systems) on 24-hour conditioned media collected from the apical and basolateral chambers of the cultured inserts as described (26). Samples were collected from 16 to 19 inserts and assayed in triplicate. The mean concentration values of the *TBC1D32* samples were expressed as a percentage of the control values.

### Statistics.

Statistical analyses were performed using the 2-tailed Mann-Whitney test, unpaired 2-tailed Student’s *t* test, or Fisher’s exact test with GraphPad Prism 8.3 software. The number of samples per experiment and condition is indicated in the corresponding figures and legends. For all analyses, a *P* value less than 0.05 was considered significant.

### Study approval.

Clinical and genetic analyses were performed after receipt of written informed consent using approved protocols of the Montpellier University Hospital, France (ID IRB-MTP_2021_11_202100959); the Research Ethics Committee of the Fundación Jiménez Díaz University Hospital, Madrid, Spain (ID CM 06-2016 FJD); the Research Ethics Committee of the University Hospital La Fe, Valencia, Spain (ID 2019/0098); and the Institute of Molecular and Clinical Ophthalmology Basel (ID 2019-01660), in agreement with the Declaration of Helsinki. Skin biopsies and iPSC reprogramming were approved by the French National Agency for the Safety of Medicines and Health Products (Saint-Denis) (ID 2014-A00549-38). Xenopus care followed institutional guidelines (licenses A91272108 and C 91-471-102), and study protocols were approved by the institutional animal care committee CEEA 59 (Paris, France) and the Direction Départementale de la Protection des Populations (Courcouronnes, France) (APAFIS 21474-2019071210549691v2 and 32589-2021072719047904v4).

### Data availability.

All data are available in the main text or the supplemental materials. Data point values for all graphs are found in the Supporting Data Values file.

## Author contributions

The authorship order among co–first authors was determined by the chronological order that they began working on the project. BB, CB, NE, DM, IM, MP, and VK designed research studies. BB, CB, NE, DM, AD, KP, CM, KK, MQ, IPR, and MC conducted experiments. CJM and HB helped with image analysis. GGG, AC, NL, GC, SG, AP, VB, VU, MD, FB, HPNS, CA, JMM, and IM performed clinical investigations. CB, CA, JMM, CR, IM, MP, and VK performed supervision. BB, CB, IM, MP, and VK wrote the manuscript. All authors reviewed and edited the manuscript.

## Supplementary Material

Supplemental data

Supporting data values

## Version 1 09/28/2023

In-Press Preview

## Version 2 11/08/2023

Electronic publication
